# How Pore Hydrophilicity Influences Water Permeability?

**DOI:** 10.34133/2019/2581241

**Published:** 2019-02-04

**Authors:** Fang Xu, Mingjie Wei, Xin Zhang, Yang Song, Wei Zhou, Yong Wang

**Affiliations:** State Key Laboratory of Materials-Oriented Chemical Engineering, Jiangsu National Synergetic Innovation Center for Advanced Materials, and College of Chemical Engineering, Nanjing Tech University, Nanjing 211816, Jiangsu, China

## Abstract

Membrane separation is playing increasingly important role in providing clean water. Simulations predict that membrane pores with strong hydrophobicity produce ultrahigh water permeability as a result of low friction. However, experiments demonstrate that hydrophilic pores favor higher permeability. Herein we simulate water molecules transporting through interlayers of two-dimensional nanosheets with various hydrophilicities using nonequilibrium molecular dynamics. We reveal that there is a threshold pressure drop (Δ*P*_T_), exceeding which stable water permeability appears. Strongly hydrophobic pores exhibit extremely high Δ*P*_T_, prohibiting the achievement of ultrahigh water permeability under the experimentally accessible pressures. Under pressures < Δ*P*_T_, water flows in hydrophobic pores in a running-stop mode because of alternative wetting and nonwetting, thus leading to significantly reduced permeability. We discover that hydrophilic modification to one surface of the nanosheet can remarkably reduce Δ*P*_T_ by > 99%, indicating a promising strategy to experimentally realize ultrafast membranes.

## 1. Introduction

Membrane separation is playing a key role in supplying potable water to people's daily life and industry [1]. Separation performances are significantly influenced by the hydrophilicity of the membrane pores. However, there are contradictory observations between experimental and simulation studies in the influence of membrane hydrophilicity on water permeability. Experimentally, it is commonly acknowledged that membranes for applications ranging from microfiltration to reverse osmosis (RO) should have strong hydrophilicity to ensure adequate water permeabilities [2–4]. Oppositely, molecular dynamics (MD) simulations demonstrate that hydrophobic pores favor enhanced water flux [5–8]. When water is confined in subnanometer pores,* e.g.,* in nanofiltration (NF) or RO membranes [9], there would be only one or two water layers inside the pores;* i.e.,* all the water molecules are included in the boundary layer. Therefore, the interaction between water molecules and pore walls would significantly influence water transport. Hydrophilicity increases the interaction between water molecules and pore walls and influences water permeability of a membrane from two opposite sides. On one hand, the positive side, the hydrophilic interaction could increase the infiltration capillary force, helping the membrane to uptake water molecules and to increase the membrane wettability. The infiltration capillary force increases dramatically with decreasing pore sizes. When the pore size is narrowed down to the subnanometer scale, water molecules inside the membrane will have greater probability to form hydrogen bonded networks, which plays an important role for water transport in the confined environment [10]. On the other hand, the negative side, stronger interaction between water molecules and pore walls results in increased friction and consequently reduces the flow velocity [11]. Larger proportion of water molecules will interact with the pore wall as the pore size decreases, and thus the friction effect becomes more pronounced. Therefore, it is necessary and worthwhile to investigate the combination effect of these two sides on water permeability.

Specifically, the recently emerging laminated membranes established from two-dimensional (2D) materials have subnanometer interlayer gaps, which offer slit-shaped pores for water transport [12–17]. In studies of these membranes, there also exists contradiction between experimental observations and simulation results on how the pore hydrophilicity influences the permeability. In experiments, Sun* et al.* [17] reported a laminar MoS_2_ membrane exhibiting a 3- to 5-times higher water flux than the graphene oxide (GO) membrane. They attributed the improved flux to the exposure of the hydrophilic sulfur atoms in the MoS_2_ monolayer sheets. Ren* et al.* [18] fabricated ion separation membranes from nanosheets of Ti_3_C_2_T_x_ MXene and they believed the promoted water flow was due to the hydrophilic nature of Ti_3_C_2_T_x_. In contrast, by MD simulations, Wei* et al.* [19] found that the flow rate exhibited a significant enhancement between graphene layers, but the enhancement broke down while the graphene sheets were modified with hydrophilic groups. Moreover, Chen* et al.* [20] simulated water transport through GO interlayers with various concentrations of hydrophilic hydroxyl groups and found that the volumetric flux was negatively related to hydroxyl concentrations, implying that stronger hydrophilicity was unfavorable for water transport. This contradiction between experimental and simulation results confuses the understanding on the effect of pore hydrophilicity on water permeability. Therefore, identifying the origin of this contradiction is of great significance not only for deeper understanding on the role of material hydrophilicity in water transport but also for the design and preparation of ultrafast membranes.

In this work, we simulate water transport through pores constructed from 2D nanosheets with various hydrophilicities by using nonequilibrium molecular dynamics (NEMD). We reveal that strong hydrophobicity leads to high water permeability but also high threshold pressure drop (Δ*P*_T_), and only the applied pressure drop exceeds Δ*P*_T_; the high permeability can occur. For hydrophobic membranes, Δ*P*_T_'s are typically at the scale of several hundreds of MPa, far more than experimentally accessible pressure drops. This explains why we seldom experimentally observe simulation-predicted ultrahigh water permeability on hydrophobic membranes. Based on these understandings we develop a new strategy—hydrophilic modification to the outer pore surface—to efficiently reduce Δ*P*_T_ of hydrophobic membranes to the experimentally accessible scale at slight expense of permeability loss, thus enabling the experimental realization of ultrafast membranes.

## 2. Results

### 2.1. Apparent Flux and Permeability

**Figure 1:**
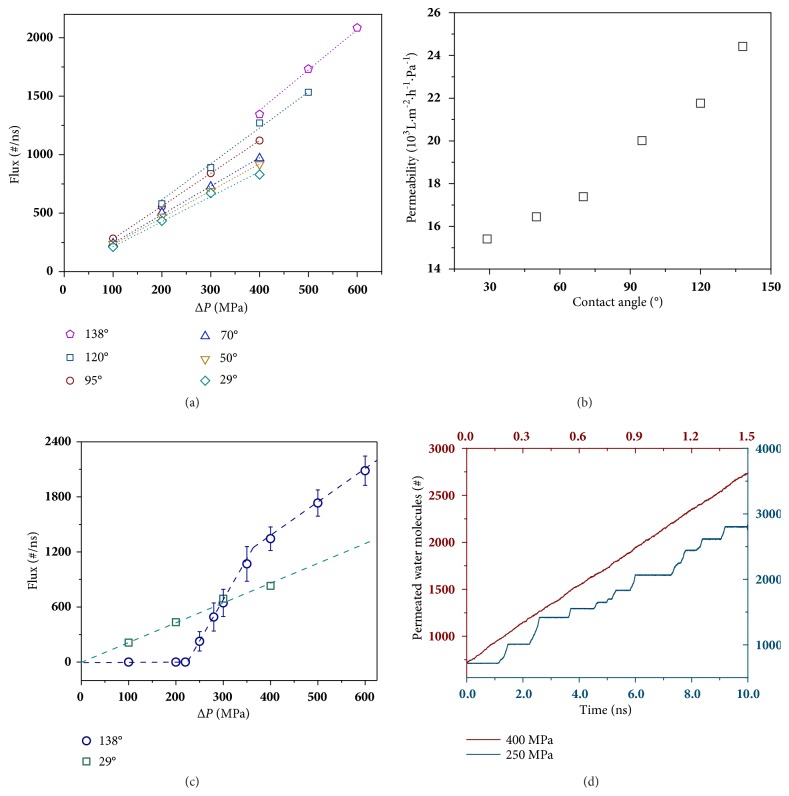
Flux and permeability. (a) Water flux and (b) permeability of membranes with various hydrophilicities represented by changing CAs. (c) Water flux of membranes with the CA of 29° and 138° within the Δ*P* range from 100 to 600 MPa. (d) Number of water molecules in the permeate side of the membrane with the CA of 138° under two typical Δ*P*s (250 and 400 MPa) as a function of simulation time.

We calculate the water flux of membranes with varying hydrophilicities under pressure drops (Δ*P*s) ranging from 100 to 600 MPa by fitting the slope of the flow curve ([Supplementary-material supplementary-material-1]). As shown in Figure 1(a), the flux of each membrane is proportional to Δ*P* despite the changing hydrophilicities. However, to achieve the continuous water flow Δ*P*s should be higher than a certain value, beyond which the proportional relationship between flux and Δ*P* is valid. For hydrophilic membranes (contact angles, CAs < 95°), the proportional relationship is valid from 100 MPa. For the membrane with CA= 120°, no water flux can be produced under Δ*P*s < 200 MPa. For the most hydrophobic membrane (CA = 138°), Δ*P* should be raised to nearly 400 MPa to obtain the continuous water flow. Apparently, these Δ*P*s are much larger than those applied in experiments. Therefore, we denote the water flux or permeability under such high Δ*P*s as apparent flux or apparent permeability as they will hardly be obtained in experiments. Figure 1(b) presents the apparent permeabilities of each membrane, which are obtained by directly fitting the flux values in Figure 1(a). It is obvious that the hydrophilicity of membranes plays a key role in governing water permeability. The permeability increases monotonically with rising hydrophobicity. Other simulation works give similar results no matter how they tune the hydrophilicity of the membrane, such as scaling the* vdW* interactions strength [21], applying artificial surface partial charge patterns [5], adjusting the density of hydroxyl groups [20], or utilizing different substances [6]. That is, all simulation works demonstrate a negative correlation between water flux and hydrophilicity.

### 2.2. Water Transport in Hydrophobic Membranes

As mentioned above, for hydrophobic membranes (CAs > 120°), no water molecule could pass through the pores and consequently no flux was measured under the Δ*P* of 100 MPa. This implies that the proportional relationship between flux and Δ*P* cannot extend to the region of lower Δ*P*s. For the most hydrophobic membrane with a CA of 138°, the proportional relationship starts around 400 MPa. However, it does not necessarily imply that it gives no flux under Δ*P*s below 400 MPa. We further simulated its flux under lower Δ*P*s with smaller pressure intervals to reveal the relationship in a broader range of Δ*P*s. As shown in Figure 1(c), it is obvious that water flux of the hydrophobic membrane (CA = 138°) is not proportional to Δ*P* and instead there exists three stages within the range of Δ*P*s from 100 to 600 MPa. In the first stage where Δ*P* < 220 MPa, no evident water flux is observed. It is found that there is no water molecule inside the membrane, indicating the nonwetting state of the membrane. In the second stage, where Δ*P* ranges from 220 to 350 MPa, the water flux rises rapidly. In the last stage, the water flux is proportional to Δ*P*s. In contrast, for the hydrophilic membrane with a CA of 29° the water flux is always proportional to Δ*P* within the entire range of Δ*P*, implying that under high Δ*P*s hydrophobic pores exhibit a wetting state similar to hydrophilic pores.

To elucidate the three-staged relationship between water flux and Δ*P* for the membrane with the CA of 138°, we investigated the microscopic details of water molecules inside this hydrophobic membrane under different Δ*P*s. We recorded the number of water molecules in the permeate side as a function of simulation time, which is shown in Figure 1(d). Sufficient water molecules were provided to the feed side so that the simulation can continue for adequate time. The evolution of the number of permeated water molecules demonstrates that the flow rate of water through the membrane is constant under 400 MPa (in the third stage), which is also similar to water passing through the membrane with a CA of 29° shown in [Supplementary-material supplementary-material-1]. However, there appears many steps in the flow curve in the case of 250 MPa (in the second stage), which implies a discontinuous water flow. This is actually a running-stop mode including alternative “running” and “stop” states.

**Figure 2:**
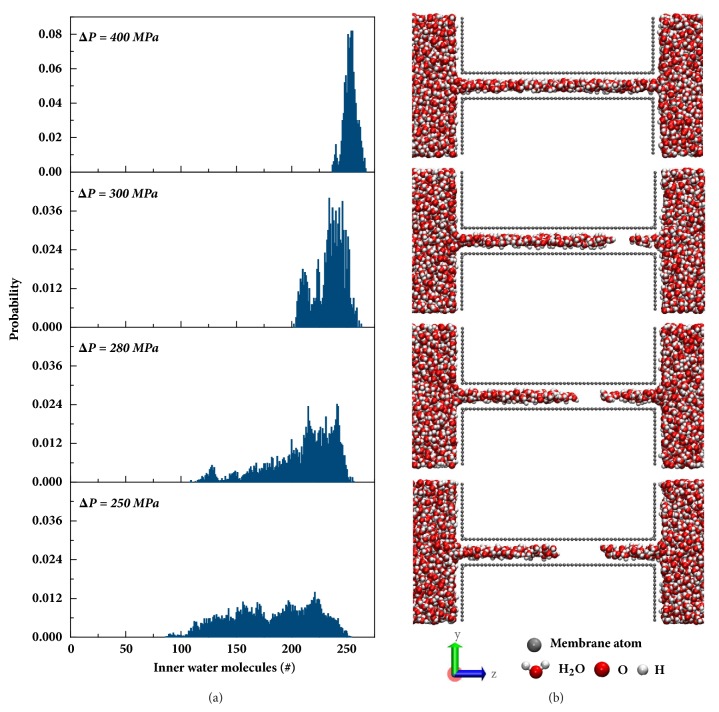
Wetting behavior of hydrophobic membranes under various Δ*P*s. (a) Probability of the number of water molecules inside the pores under different Δ*P*s. (b) Representative snapshots of the water stream inside the pore during the flowing process under the corresponding Δ*P*. The water molecules are presented in red (oxygen atom) and white (hydrogen atom). The membranes are colored in grey.

To quantitatively investigate this “running-stop” water flow, we monitored the number of water molecules inside the membrane (*N*_water_) during the entire flow process and revealed the tendency of its variations.  Figure 2(a) illustrates the probability distribution of *N*_water_ under four different Δ*P*s spanning from the second to the third stage. In the case of 400 MPa, *N*_water_ is distributing near 250, indicating that the number of water molecules remains ~250 during the simulation time. Nevertheless, when Δ*P* is decreased from 400 MPa to 250 MPa, *N*_water_ distributes in an increasingly wide range, indicating that the pores are not completely filled with water during the entire simulation time. As vividly shown in the snapshots in Figure 2(b), water streams inside the pores under these Δ*P*s break off during the flowing process. This results in water transport in the running-stop mode.

When the system reaches a steady state, the driving force and resistance are in balance, which leads to a continuous water flow and a stable flux. Oppositely, the running-stop transport is a metastable state with a wetting and a nonwetting state repeating alternately rather than a stable continuous flow. The hydrogen bonded network that stretched from the entrance to the exit of the pore was reported to be responsible for the fast transport in membranes made of 2D grapheme [12]. Moreover, the hydrogen bonded network must be reformed when water molecules enter highly confined pores [22, 23]. Based on these understandings, we realize that in the second stage the wetting behavior of water molecules cycles between the wetting state and the nonwetting state. In the wetting state, the hydrogen bonded network completely forms, therefore, the running state is reached and the flow occurs. However, in the nonwetting state, the water stream is disconnected as the hydrogen bonded network breaks, terminating water flow (the stop state).  Figure 2(a) shows *N*_water_ distributes in wider ranges with decreasing Δ*P*s, indicating the running state lasts shorter and the stop state last longer. Consequently, lower water permeabilities are obtained under decreasing Δ*P*s. When the Δ*P* is decreased to be lower than 220 MPa, the stop states (nonwetting) dominate, and consequently no flux can be observed. Similarly, the transport mode would turn into the constant running state (wetting) when the Δ*P* rises to a certain value, beyond which the water flux becomes always proportional to Δ*P*s.

### 2.3. The Threshold Pressure Drop

**Figure 3:**
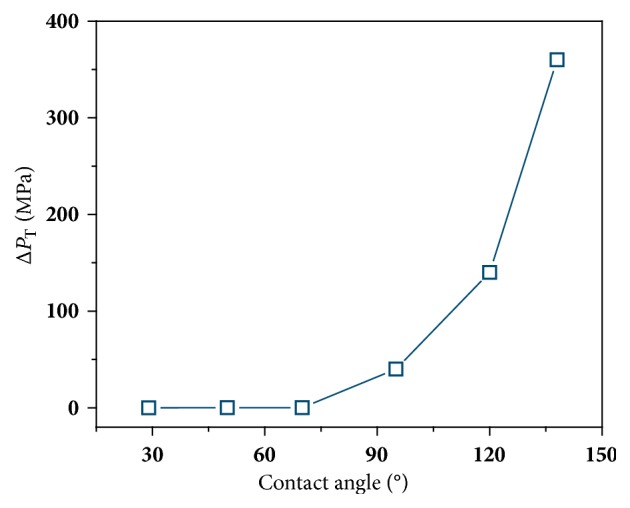
Threshold pressure drops (Δ*P*_T_'s) for membranes with various hydrophilicities indicated by CAs increased from 29° to 138°.

We term this critical Δ*P*, above which the pores are in the wetting state and water flow reaches the constant running state, the threshold pressure drop, Δ*P*_T_. The Δ*P*_T_'s for membranes with various CAs are plotted in Figure 3. For hydrophilic membranes (CA = 29°, 50°, or 70°), the Δ*P*_T_'s are nearly zero because these hydrophilic membranes could be constantly wetted and no external pressure is needed. For hydrophobic membranes, the Δ*P*_T_ appears and rises rapidly with increasing CAs. Now we understand that higher hydrophobicity leads to greater water permeability but higher Δ*P*_T_'s. Δ*Ps* used in experimental works generally exceed Δ*P*_T_'s of hydrophilic membranes, so one could easily obtain the continuous flux. For hydrophobic membranes, the Δ*P*_T_'s are much higher than experimental pressures, and it is difficult to wet the pores under the experimentally used Δ*P*s, resulting in no water permeability. In contrast, in simulations the employed Δ*P*s are typically in the scale of several hundreds of MPa, generally exceeding the Δ*P*_T_'s of both hydrophobic and hydrophilic membranes, and both membranes can reach the wetting state. Once wetted, hydrophobic membranes exhibit higher water permeabilities than hydrophilic ones due to the low friction of the former, as shown in Figure 1(b). This explains the contradictory results between experiments and simulations with respect to the influence of pore hydrophilicity on the water permeability.

### 2.4. Modification Strategies to Reduce Threshold Pressure Drop

Hydrophobic membranes exhibit ultrahigh water permeabilities, but only under extremely high pressures because of their high Δ*P*_T_'s. Therefore, to experimentally realize the ultrahigh permeabilities, the Δ*P*_T_'s of hydrophobic membranes should be significantly depressed to the scale close to the experimentally used Δ*P*s. Δ*P*_T_'s are dependent on the wetting state of the membrane. Therefore, suitably changing the wetting behavior of the membranes by hydrophilic modification may reduce Δ*P*_T_'s at no or little expense of water permeabilities. In addition to the complete hydrophilic modification to pore walls, which totally eliminates the pristine hydrophobicity of the pores and consequently the ultrahigh permeability, there has emerged a few other strategies to perform hydrophilic modification on selective positions of 2D nanosheets, that is, hydrophilic modification to the pore entrances [24] and regionally hydrophilic modification inside the pores [12, 25]. We then examine their efficiency in reducing Δ*P*_T_'s.

**Figure 4:**
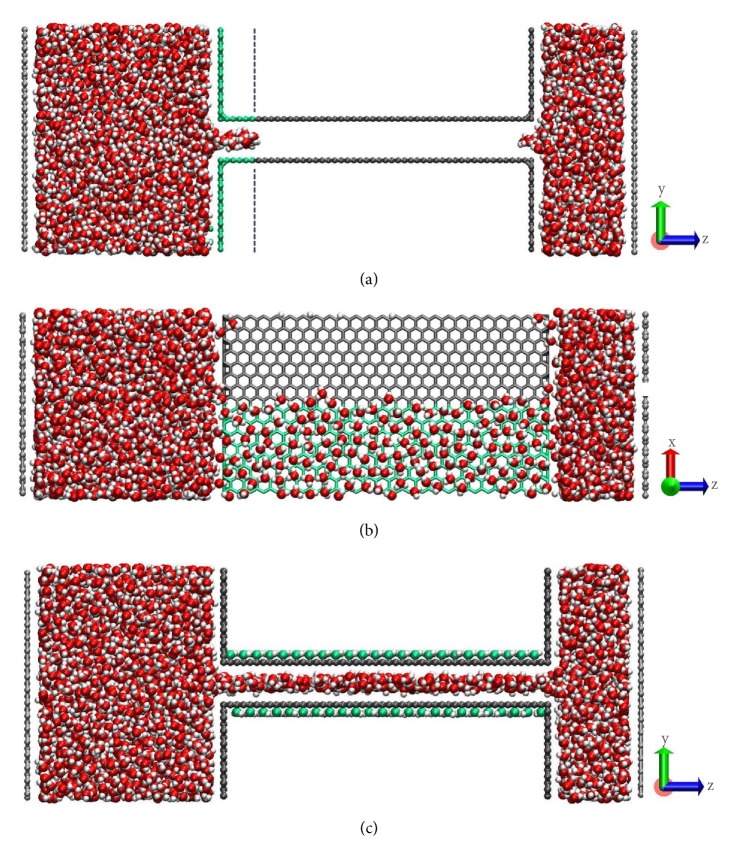
Three simulation models and the wetting states of hydrophobic membranes subjected to different hydrophilic modifications under Δ*P *=1 MPa. (a) Hydrophilic modification to the pore entrances; (b) regionally hydrophilic modification inside the pore; (c) hydrophilic modification to the outer pore walls. Green and dark grey represent the hydrophilic and hydrophobic part, respectively, and oxygen and hydrogen atoms in hydroxyl groups are marked in green and white, respectively.

In the first strategy, the hydrophilic modification was applied only to the entrance region while the inner pore was still kept its pristine hydrophobicity (with CA of 95°, 120°, or 138°). The entrance region was defined as 5 Å from the inlet of the pore and modified with atoms to give a CA of 29°. We investigated the wetting behavior and the permeability under both low and high Δ*P*s. As shown in Figure 4(a), water molecules could occupy only the modified part at the entrance region under low Δ*P*s (≤1 MPa), suggesting that the Δ*P*_T_ exists not only at the entire entrance region, but throughout the inner hydrophobic area. As a result, we did not observe any water molecules passing through the membranes. Under high Δ*P*s (hundreds of MPa), the water flux also experience the three-staged flow as described above. Moreover, no evident change of Δ*P*_T_ for these membranes with various CAs is observed. The ∆*P*_T_ in this case is still as high as that for the unmodified hydrophobic membrane, indicating that this hydrophilic modification takes little effect on depressing the Δ*P*_T_.

Hydrophilic modification to graphene is frequently achieved by oxidation [26], and the generated oxygen-containing groups prefer to form clusters, which resulting in the patches of pristine (hydrophobic) and oxidized (hydrophilic) regions on the surface of grapheme [27–29]. This can be considered as regionally hydrophilic modification inside the pore. To find out the influence of this modification on water permeability, the pore wall was patterned with two regions: one-half is hydrophilic (CA = 29°) and the other is hydrophobic (CA = 95°, 120°, or 138°). Under low Δ*P*s, there are two distinctive wetting states for membranes with different hydrophilicities. When the hydrophobicity is moderate (CA = 95°), water molecules start to occupy the hydrophilic region and then saturate the hydrophobic region progressively, indicating that the impact of hydrophilic interaction help to uptake more water molecules into the pores. Ban* et al.* [30] also observed that water first saturated the oxidized regions and then flooded to the pristine regions in GO membranes. Δ*P*_T_ of this modified membrane is significantly decreased from ~ 40 MPa for the pristine membrane to less than 0.1 MPa, while the permeability under high Δ*P*s is moderately decreased from ~20×10^3^ to ~17×10^3^ L·m^−2^·h^−1^·MPa^−1^.

However, as can be seen from Figure 4(b) when the CA of the hydrophobic region is higher than 120°, water molecules would not wet the hydrophobic region after covering the hydrophilic region. This implies that the Δ*P*_T_ of the hydrophobic region cannot be depressed by this regional modification. The nonwetting state of the hydrophobic regions in the pores means that the actual porosity under this condition turns into only half of the pristine one, thus, the flux will not be higher than half of the pristine one. While under high Δ*P*s, water molecules fill the membrane completely like that in the unmodified hydrophobic membrane, however the water permeability is lower compared to the unmodified hydrophobic membrane. Wei* et al.* [25] attributed this to a prominent side-pinning effect. Due to the inconsistence of wetting under low and high Δ*P*s, it is unreliable to extrapolate the permeability under high Δ*P*s to those under low Δ*P*s. Therefore, we understand that the regional hydrophilic modification cannot depress Δ*P*_T_'s of strongly hydrophobic membranes although it works for moderately hydrophobic membranes (CA < 120°).

Alternatively, we propose another strategy, that is, hydrophilic modification to the outer pore walls, which can efficiently reduce Δ*P*_T_'s of strongly hydrophobic membranes (CA = 120° or higher). The fast water transport through graphene interlayers is prohibited by the hydrophilic oxygen-containing groups inside the pores [19, 20]. This is because the interaction between water and hydrophilic groups is too strong. Considering that 2D monolayer nanosheets are only one-atom thick, we anticipate that presence of these groups on the outer surface will reduce the interaction to some degree but will not fully eliminate it. Thus, the modification to the outer walls may also affect the water transport inside the pore across this one-atom thickness. As shown in Figure 4(c), the commonly used hydrophilic hydroxyl groups are chosen to functionalize the outer walls. The hydroxyl groups were randomly distributed outside the pore wall with concentration* c* = *n*_OH_/*n*_C_ = 24%, which is similar to both experimental [31, 32] and simulation [20] results. The all-atom optimized potential for liquid simulations (OPLS-AA) [33] was used for the functional groups, which is successfully applied in previous studies on the graphene oxides membrane [19, 25, 34].

**Figure 5:**
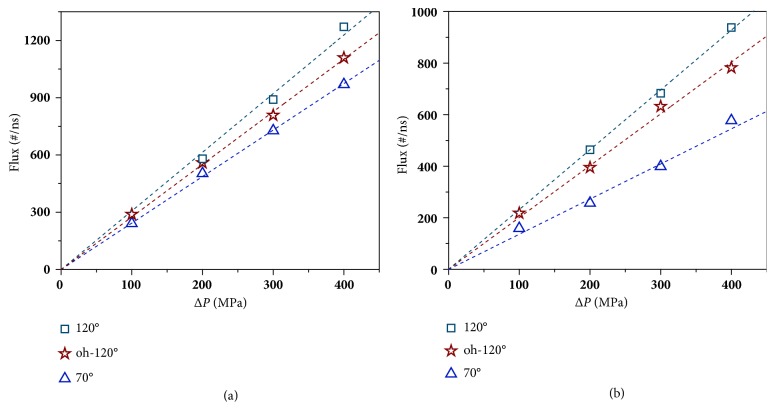
Flux of the (a) 0.8 nm-wide and (b) 0.7 nm-wide pores with different CAs. “oh-120°” indicates the membrane with a CA of 120° for the inner pore surface and the outer pore surface subjected to hydrophilic modification.

We investigated the membrane with the CA of 120°, whose Δ*P*_T_ cannot be reduced by the strategy of regional modification as we discussed above. By hydrophilic modification to the outer surface the pore could be wetted under low pressures (~1 MPa), suggesting that the Δ*P*_T_ is depressed over 99% as it is ~140 MPa for the pristine membrane. Meanwhile, the permeability under high Δ*P*s, which is the slope of the flux-Δ*P* curve, is decreased by only 10 % (Figure 5(a)) and this value could be applied to the low Δ*P*s due to the consistence of their wetting states. Moreover, to exclude the particularity of the channel size, we also applied this method to 0.7 nm-wide pores, and obtain similar results as shown in Figure 5(b). Furthermore, Figure 5 also indicates that thus modified membranes exhibit much higher water permeability than initially hydrophilic membranes, for instance, the membrane with a CA of 70°. Therefore, we conclude that hydrophilic modification to the outer pore surface is a highly efficient strategy to reduce Δ*P*_T_ at slight expense of water permeability. This modification strategy requires hydrophilic modification exclusively on one side of 2D monolayers while keeps the strong hydrophobicity of the other side intact. Importantly, such a “Janus” 2D structure has already been experimentally obtained [35, 36], implying the big potential to experimentally realize ultrafast membranes by this hydrophilic modification strategy.

## 3. Discussion

In summary, membranes were constructed from 2D nanosheets with varying hydrophilicities to investigate the contradiction between experimental and simulation results on the influence of the pore hydrophilicity on water transport. We discover that the contradiction originates from the discrepancy of pressure drops (Δ*P*) in experiments and simulations. For hydrophilic membranes, there is a proportional relationship between flux and Δ*P*s. In contrast, for hydrophobic membranes, this proportional relationship only exits under high Δ*P*s. Under a threshold pressure drops, Δ*P*_T_, there is no flux or a discontinuous water flow in the “running-stop” mode. In simulations, the Δ*P* is usually set to be extremely high to accelerate the computing. This high Δ*P* could facilely exceed the Δ*P*_T_, so that the membranes are always in the wetting state. The different wetting states hinder the extrapolation of permeability from high Δ*P*'s to low Δ*P*'s, leading to the contradictory understanding on the effect of hydrophilicity. Unlike the hydrophobic membranes, the hydrophilic membranes could be wetted under low Δ*P*s, indicating Δ*P*_T_ is nearly zero. We investigate the efficiency of different hydrophilic modifications in depressing Δ*P*_T_'s of hydrophobic membranes and propose a new strategy, hydrophilic modification of the outer pore wall, which is able to depress Δ*P*_T_'s by > 99% of highly hydrophobic membranes at the slight expense of ~ 10% reduction in water permeability. This work identifies the origin of the contradictions between experimental and simulation results on the effect of pore hydrophilicity on water permeability, which not only helps to understand water transport in nanopores but also to design and experimental realization of ultrafast membranes.

## 4. Methods

The simulation system consisted of a membrane with one water reservoir and one additional moveable piston at each end, as shown in [Supplementary-material supplementary-material-1]. A 2D nanosheet bilayer was set to be parallel to the* yz* plane and modeled as the slit pore, and the length of the pore was set to 6 nm while the thickness of the pore was set to 0.8 nm, which was based on the interlayer spacing of the graphene oxide membrane [12, 31, 37]. Another four constraint walls (vertical to the pore wall and parallel to the* xy* plane) were served as the membrane surfaces, which were used to embed the pores and restrict the movement of water molecules. Such a membrane model is reliable and has also been adopted in other MD simulations [21]. The two water reservoirs formed the feed side (high pressure) and the permeate side (low pressure). Hence, the flow direction through the membrane was defined along the* z* direction. Besides, the system was bounded by two rigid pistons used to create a pressure drop (Δ*P*) across the membrane. The simulation box lengths in* x* and* y* directions are set equal to the lateral size of the bilayer (34.3 Å) and the height of four constraint walls (43.7 Å), respectively.

All the simulations were carried out using the large-scale atomic/molecular massively parallel simulator (LAMMPS) package. The SPC/E model was utilized to construct the water molecules. The SHAKE algorithm was applied to constrain the bonds and angles of the water molecules, which reduced high frequency vibrations and saved the simulation time. The interaction for all atoms included* vdW* and electrostatic terms. The former was modeled with a LJ potential, 4*ε*[(*σ*/*r*)^12^-(*σ*/*r*)^6^], and the cutoff was set as 1.0 nm. In this work, the structure of graphene was merely used as the channel model. According to Werder* et al*. [38], the hydrophilicity of the membrane could be tuned by changing the* ε* parameter between C and O atoms while keeping the* σ* parameter fixed. The* ε *parameter signifies a microscopic interaction, which is not a macroscopic manifestation of hydrophilicity. In addition, the CA of a water droplet on a surface is usually employed to measure the membrane hydrophilicity, which is a macroscopic manifestation of the microscopic interactions between the surface and the water molecules. Hence, according to the work studying the CA of water on 2D planes with varying* ε* parameters [38], we also adopt the CAs ranging from 29° to 138° to characterize and distinguish the membranes with varying hydrophilicities. This method to adjust hydrophilicity was reported to have a negligible effect on the channel diameter [39]. The long-range electrostatic interactions are computed by using the particle-particle particle-mesh (PPPM) algorithm with a root mean square accuracy of 10^−5^. The membrane models were fixed in position throughout the simulation, and periodic boundary conditions (PBC) were applied in the* x *and* y *directions. A time step of 1 fs was used for all simulations.

Initially, for each simulation, the energy of system was minimized for 1000 steps. Then an external force along the -z/+z direction,* f*, is applied to each atom of top/bottom piston to produce a pressure of 1 atm (Equation (1)), allowing the system to reach the desired pressure and the bulk water to reach the equilibrium density (1 g/cm^3^).

(1) $$\begin{array}{c} f=\frac{PA}{n} \end{array}$$

where* P* denoted the desired pressure on the piston,* A* was the area of the piston, and* n* represented the atom number of the piston. The system was kept at room temperature (300K) using the Berendsen thermostat. After the equilibrium simulation of 2 ns, the NEMD simulations were carried out by applying another external force on the bottom piston, so the Δ*P* could be calculated by

(2) $$\begin{array}{c} ∆P=P_{bottom}-P_{top} \end{array}$$

After the first 1 ns of stabilization of the NEMD simulation, statistics and trajectories were gathered during 2-10 ns for different systems, which was long enough to obtain the well-converged simulation results. This method of applying Δ*P* has been thoroughly described in a previous study [40] and adopted by many other researchers to simulate the membrane process [41–44]. To enhance the signal-to-noise ratio and accelerate the NEMD simulations, like other simulating studies [41–46] we perform the simulations under very high Δ*P*s (100 MPa-600 MPa), which are much higher than pressures applied in experiments and industrial usage.

## Supplementary Materials

Supplementary Materials
** Calculation of water flux by fitting the slope of the flow curve.** The numbers of water molecules in the feed side, inside the pores, and in the permeate side were tracked over simulation time. To justify the steady state of the system, Figure S1 shows an exemplificative evolution of numbers of water molecules in the three phases over the sampling period for the membrane with a 0.8 nm-wide pore and a contact angle of 29°. In Figure S1, the blue region represents the numbers of water molecules in feed side, while the grass green region represents those in permeate side. The number of water molecules in feed side drops linearly as a function of simulation time; at the same time, the number for permeate side rises linearly. The dark green region represents the number of water molecules in the inner part. The height of this part is unchanged, indicating the constant number of water molecules in the inner part. Besides, the linear relationship indicates that water molecules permeate through the membrane at a constant rate. Therefore, the slope of the flow curve in Figure S1 corresponds to the water flux.
