# Supplementary material for: How Pore Hydrophilicity Influences Water Permeability?
Source: Research (Wash D C). 2019 Feb 4;2019:2581241. doi: 10.34133/2019/2581241 (PMC6750107; doi:10.34133/2019/2581241)
Supplement: Supplementary Materials — Calculation of water flux by fitting the slope of the flow curve. The numbers of water molecules in the feed side, inside the pores, and in the permeate side were tracked over simulation time. To justify the steady state of the system, Figure S1 shows an exemplificative evolution of numbers of water molecules in the three phases over the sampling period for the membrane with a 0.8 nm-wide pore and a contact angle of 29°. In Figure S1, the blue region represents the numbers of water molecules in feed side, while the grass green region represents those in permeate side. The number of water molecules in feed side drops linearly as a function of simulation time; at the same time, the number for permeate side rises linearly. The dark green region represents the number of water molecules in the inner part. The height of this part is unchanged, indicating the constant number of water molecules in the inner part. Besides, the linear relationship indicates that water molecules permeate through the membrane at a constant rate. Therefore, the slope of the flow curve in Figure S1 corresponds to the water flux. [file 2581241.f1.zip › 2581241.f1/Fangfigs2.docx]

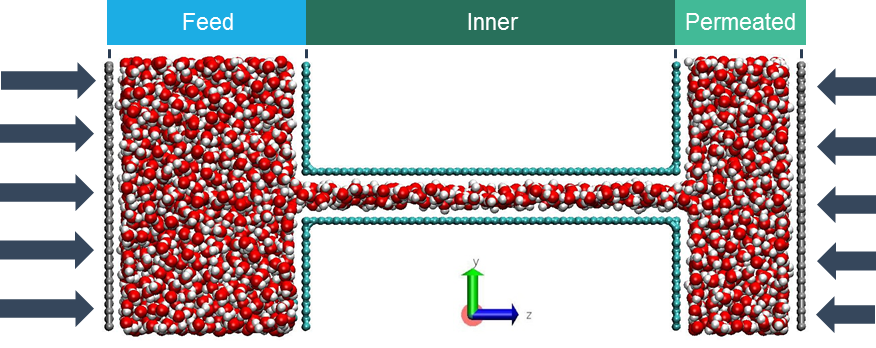


Fig. S2. Model diagram of the simulation system. The water molecules are presented in red (oxygen atom) and white (hydrogen atom). The membrane and pistons are colored in cyan and grey, respectively.
